# Supplementary material for: Patterns of Intron Gain and Loss in Fungi
Source: PLoS Biol. 2004 Nov 30;2(12):e422. doi: 10.1371/journal.pbio.0020422 (PMC532390; doi:10.1371/journal.pbio.0020422)
Supplement: Table S1 — Also available at http://genes.mit.edu/NielsenEtAl/. (4.3 MB ZIP). [file pbio.0020422.st001.zip › NielsenEtAl/html/591.html]

AN5210.1.NCU06075.1.MG08063.1.FG07528.1


```
 CLUSTAL W (1.82) Multiple Sequence Alignments - Introns Inserted


Sequence 1: MG08063.1	528 aa
Sequence 2: FG07528.1	540 aa
Sequence 3: NCU06075.1	527 aa
Sequence 4: AN5210.1	526 aa
Alignment Length: 541 aa
Number Identitical Residues: 320 aa
Alignment Score (without introns) 14905


MG08063.1 	-------------MATALDHLYIGGRINWLAQLNTAAQPDKNYRRSSIICTIG~PKTNSV
NCU06075.1	------------MAATALDHLSLGGKIQWLAQLNTEFQPAREFRRTSIICTIG~PKTNSV
FG07528.1 	MPQKSENQNKSVMATTAQDHLEFGGKISWLASLDTAFRPERNYRRSSIICTIG~PKTNSV
AN5210.1  	-----------MAASSSLDHLSNRMKLEWHSKLNTEMVPAKNFRRTSIICTIG1PKTNSV
          	              ::: ***    ::.* :.*:*   * :::**:******* ******

MG08063.1 	EAMNDLRKA~GMNVVRMNFSHGSYE~YHQ~SVIDNAREAEKQMPGRQLAIALDT~KGPEI
NCU06075.1	EAINKLRDA~GLNVVRMNFSHGSYE~YHQ1SVIDNARQAEKVHPGRPIAIALDT~KGPEI
FG07528.1 	EAINKLRDS~GLNVVRMNFSHGSYE~YHK~SVIDHARESEATHAGRNVAIALDT~KGPEI
AN5210.1  	EKINALRRA1GLNVVRMNFSHGSYE0YHQ~SVIDHAREAEKQQAGRPVAIALDT0KGPEI
          	* :* ** : *:************* **: ****:**::*   .** :****** *****

MG08063.1 	RTGNTKNDEDLPISAGKELNFTTDEQYATSCDTDNV2YVDYKNITKVIEKGRIIYVDDGV
NCU06075.1	RTGNTKNDEDIPISAGTILNITTDEKYKDECTIEHM2YVDYVNITKVIAPGRIIYVDDGV
FG07528.1 	RTGNTPGDEDIPISVGHEMNITTDDAYATASDDKNM2YVDYKNITNVIEPGRIIYVDDGV
AN5210.1  	RTGNTVGDKDIPIKAGHEMNISTDEQYATASDDQNM2YVDYKNITKVISAGKLIYVDDGI
          	***** .*:*:**..*  :*::**: *   .  .:: **** ***:**  *::******:

MG08063.1 	LAFEVLDVIDEKTVRVRARNNGFICSKKGVNLPNTDVDLPALSEKDKADLRFGVKNNVDM
NCU06075.1	LAFEVLEIVDDKTIKVKARNNGYISSRKGVNLPNTDVDLPALSEKDKADLRFGVKNKVDM
FG07528.1 	LAFDVLKIVDEKTILVKARNNGAICSKKGVNLPNTDVDLPALSEKDKADLKFGVENNVDM
AN5210.1  	LSFEVLEVVDDKTLRVRCLNNGNISSRKGVNLPGTDVDLPALSEKDISDLKFGVKNKVDM
          	*:*:**.::*:**: *:. *** *.*:******.************ :**:***:*:***

MG08063.1 	VFAS~FIRRGQDIKDIREVLGQDGAHIQIIAKIENRQGLNNFPEILKETDGVMVARGDLG
NCU06075.1	VFAS1FIRRGQDIKDIREVLGEDGKQIQIIAKIENRQGLNNFAEILAETDGVMVARGDLG
FG07528.1 	VFAS~FIRRAQDIYDIRDVLGEQGKRIQIIAKIENRQGLNNFREILEATDGVMVARGDLG
AN5210.1  	VFAS~FIRRGSDIRHIREVLGEEGREIQIIAKIENQQGVNNFDEILEETDGVMVARGDLG
          	**** ****..** .**:***::* .*********:**:*** ***  ************

MG08063.1 	IEIPAAEVFAAQKKMIALCNMAGKPVICATQMLESMIKNPRPTRAEISDVGNAVTDGSDC
NCU06075.1	IEIPAAEVFAAQKKIIAMCNIAGKPVICATQMLESMIKNPRPTRAEISDVGNAVTDGADC
FG07528.1 	IEIPAAEVFAAQKKLIAMCNLAGKPVICATQMLESMIKNPRPTRAEISDVGNAITDGADC
AN5210.1  	IEIPAPKVFIAQKMMIAKCNIKGKPVICATQMLESMTYNPRPTRAEVSDVANAVLDGADC
          	*****.:** *** :** **: **************  ********:***.**: **:**

MG08063.1 	VMLSGETAKGSYPCEAVREMSDACLKAENTIPYVSHFEEMCGAVHRPVSIVESCAMAAVR
NCU06075.1	VMLSGETAKGAYPTEAVREMSEAVLKAENTIPYVSHFEELCSLAKRPVSIVESCAMASVR
FG07528.1 	VMLSGETAKGSYPSEAVKEMHEACLKAENTIPYVSHFEEMCTLVKRPVSTVESCAMAAVR
AN5210.1  	VMLSGETAKGNYPCEAVTMMSETCLLAEVAIPHFNVFDELRNLAPRPTDTVESIAMAAVS
          	********** ** ***  * :: * ** :**:.. *:*:   . **.. *** ***:* 

MG08063.1 	ASLDINAGGIIVLSTS~GVSARLLSKYRPVCPIFMVTRNASASR~YSHLYRGVYPFLFPE
NCU06075.1	ASLDLNAAAILVLSTS~GESARLISKYRPVCPIIMITRNDSASR~YAHLYRGVYPFLFPE
FG07528.1 	ASLDLGAGGIIVLSTS~GESARMLSKYRPVCPIFMVTRSPTTSR~FAHLYRGVYPFLFPE
AN5210.1  	ASLELNAGAIVVLTTS2GNTARMISKYRPVCPIIMVSRNPAATR0YSHLYRGVWPFYFPE
          	***::.*..*:**:** * :**::*********:*::*. :::* ::******:** ***

MG08063.1 	AKPDFSKVNWQEDVDRRIKWGVKHALDLGVLEKNDSIVVVQGWKGGMGNTNTIRVLKADT
NCU06075.1	SKPDFSKVNWQEDVDRRIKWGLSHGIGLKVLNEGETVVVVQGWKGGMGNTNTFRIVKADV
FG07528.1 	QKPDFAQVNWQEDVDRRIKWAVNNALELNVLTPGDTVVVVQGWKGGMGNTNTLRIVKADP
AN5210.1  	KKPDFNVKIWQEDVDRRLKWGINHGLKLGIINKGDNIVCVQGWRGGMGHTNTVRVVPAEE
          	 ****    ********:**.:.:.: * ::  .:.:* ****:****:***.*:: *: 

MG08063.1 	ESLGIGQADI
NCU06075.1	NHLGLGQP--
FG07528.1 	EHLGIGQLQ-
AN5210.1  	N-LGLSE---
          	: **:.:
```
